# Draft Genome Sequence of the Termite-Associated “Cuckoo Fungus,” *Athelia* (*Fibularhizoctonia*) sp. TMB Strain TB5

**DOI:** 10.1128/MRA.01230-20

**Published:** 2021-01-07

**Authors:** Zachary Konkel, Kelsey Scott, Jason C. Slot

**Affiliations:** a Center for Applied Plant Sciences, The Ohio State University, Columbus, Ohio, USA; b Department of Plant Pathology, The Ohio State University, Columbus, Ohio, USA; Broad Institute

## Abstract

Atheliales is a diverse order of crust-forming Basidiomycota fungi. Here, we report the draft genome of the “cuckoo fungus,” *Athelia* (*Fibularhizoctonia*) sp. TMB strain TB5 (Atheliales), which forms termite-egg-mimicking sclerotia that termites tend. We further compare its repertoire of psilocybin gene homologs to homologs previously reported for *Fibularhizoctonia psychrophila*.

## Abstract (precis)

Atheliales is a diverse order of crust-forming Basidiomycota fungi. Here, we report the draft genome of the “cuckoo fungus,” *Athelia* (*Fibularhizoctonia*) sp. strain TMB (Atheliales), which forms termite-egg-mimicking sclerotia for which termites care. We further compare its repertoire of psilocybin gene homologs to homologs previously reported for *Fibularhizoctonia psychrophila*.

## ANNOUNCEMENT

Atheliales (Agaricomycetes, Basidiomycota) is an ecologically diverse (1–4) order of resupinate fungi. Two Atheliales genomes were previously reported, namely, *Fibularhizoctonia psychrophila* CBS 109695, associated with carrot spoilage (2, 5), and *Piloderma olivaceum* F 1598, an ectomycorrhizal tree associate (4). *F. psychrophila* has close homologs of genes in the Agaricales (Agaricomycetes) psilocybin gene cluster (6).

*Athelia* (*Fibularhizoctonia*) sp. TMB (Atheliales) produces sclerotia (termite balls [TMB]) that chemically and structurally mimic *Reticulitermes* sp. eggs and receive care in over 70% of colonies of some *Reticulitermes* species (7–9). Although this is sometimes considered parasitism (9), reciprocal benefits of the symbiosis are being investigated (7, 10). The *F. psychrophila* genome previously reported is not termite associated (4).

*Athelia* sp. TMB TB5 sclerotia were obtained in July 2017 from a *Reticulitermes flavipes* colony at the Denison Bioreserve (Granville, OH, USA). Sclerotia were cultured in potato dextrose broth at room temperature for 14 days with shaking. Tissue was filtered through Miracloth, flash-frozen with liquid nitrogen, and pulverized with a mortar and pestle. Genomic DNA was immediately extracted using the DNeasy plant minikit (Qiagen). Short-read DNA libraries were prepared using the NEBNext Ultra DNA library preparation kit and were sequenced with a 150-bp paired-end format on a NovaSeq 6000 system (Illumina). Long reads were generated by fragmenting genomic DNA with a Covaris g-TUBE, preparing libraries using the SQK-LSK108 ligation sequencing kit, and sequencing the libraries on a MinION system (Oxford Nanopore Technologies) using an R9.4 flow cell. Sequencing generated 62,281,267 Illumina reads with an average coverage of 230.98× and 228,721 MinION reads with a mean length of 3,983.38 bp, a median length of 1,834 bp, and a mean coverage of 10.94×. Coverage was calculated by mapping reads to the assembly with Bowtie 2 v2.4.1-2 (11) and minimap2 v2.2.17 (12).

Illumina reads were trimmed using Trimmomatic v0.36 (13) with the following parameters: PE-phred33, ILLUMINACLIP TruSeq3-PE.fa:2:30:10:11, HEADCROP 10, CROP 145, SLIDINGWINDOW 50:25, and MINLEN 100. MinION reads were base called with Guppy v3.0.3 (Oxford Nanopore Technologies). Hybrid assembly was conducted using SPAdes v3.12.0 (14) with auto coverage cutoff and kmers 21, 33, 55, 77, 99, and 121. Repeat elements were identified *de novo* using default parameters with RepeatModeler v1.0.11 (15) and the Dfam v3.0 repeat library (16). Genes were predicted using SNAP v2006-07-28 (17), AUGUSTUS v3.3.3 (18), GlimmerHMM v3.0.4 (19), and GeneMark-ES v4.35 (20) via the Funannotate v1.7.4 pipeline (21). We referenced the *Laccaria bicolor* benchmarking universal single-copy ortholog (BUSCO) data set (22), *F. psychrophila* expressed sequence tag (EST) and transcript data (4), and protein data from 11 Agaricomycetes MycoCosm records (Joint Genome Institute [JGI]), i.e., *F. psychrophila* CBS 109695, *P. olivaceum* F 1598 (https://mycocosm.jgi.doe.gov/Pilcr1), *Coniophora olivacea* MUCL 20566 (https://mycocosm.jgi.doe.gov/Conol1), *Coniophora puteana* (https://mycocosm.jgi.doe.gov/Conpu1), *Paxillus involutus* ATCC 200175 (https://mycocosm.jgi.doe.gov/Paxin1), *Pisolithus microcarpus* 441 (https://mycocosm.jgi.doe.gov/Pismi1), *Pisolithus tinctorius* Marx 270 (https://mycocosm.jgi.doe.gov/Pisti1), *Rhizopogon vesiculosus* Smith (https://mycocosm.jgi.doe.gov/Rhives1), *Scleroderma citrinum* Foug A (https://mycocosm.jgi.doe.gov/Sclci1), *Serpula himantioides* MUCL 38935 (https://mycocosm.jgi.doe.gov/Serla_varsha1), and *Suillus brevipes* Sb2 v2.0 (https://mycocosm.jgi.doe.gov/Suibr2). Gene prediction was finalized using OrthoFiller v1.1.1 (23) with default parameters referencing nine Agaricomycetes species, i.e., *Armillaria gallica* 21-2 (https://mycocosm.jgi.doe.gov/Armga1), *C. olivacea* MUCL 20566, *F. psychrophila* CBS 109695, *Galerina marginata* (https://mycocosm.jgi.doe.gov/Galma1), *Laccaria bicolor* v2.0 (https://mycocosm.jgi.doe.gov/Lacbi2), *P. croceum* F 1598, *Plicaturopsis crispa* (https://mycocosm.jgi.doe.gov/Plicr1/Plicr1.home.html), *Psilocybe serbica* (https://mycocosm.jgi.doe.gov/Psiser1), and *S. brevipes* Sb2 v2.0.

Average amino acid identity (AAI) between *F. psychrophila* and *Athelia* sp. TMB TB5 was calculated from the BLASTp (24) identity of all 1,727 single-copy orthologs predicted using OrthoFinder v2.3.8 (25) with *P. olivaceum, F. psychrophila, and Athelia* sp. TMB TB5 and default parameters. Sequences similar to *Psilocybe cubensis* psilocybin gene sequences (26) were obtained via BLASTp searches against NCBI and JGI fungal proteomes. Core psilocybin Pfam (27) domains were extracted from BLAST hits with greater than 50% Pfam coverage (hmmsearch v3.1b2 [hmmer.org]). Protein domains were aligned (MAFFT v7.467 [28] with --auto specified), trimmed (trimAl v1.4.rev15 [29], -automated1 specified in iteration 1 and -gt 0.25 specified in iteration 2), and used to construct maximum likelihood phylogenies (1,000 bootstraps; IQ-TREE v2.0.3 [30]). Close homologs of psilocybin proteins were those located in the largest clade that included only the psilocybin cluster (6) and Atheliales sequences.

*Athelia* sp. TMB TB5 has a mean AAI of 82.58% (median, 93.95%) with *F. psychrophila* across 1,727 single-copy orthologs, consistent with substantial divergence time. Multiple copies of psilocybin decarboxylase, hydroxylase, and kinase in *Athelia* sp. TMB TB5 (Table 1) indicate their broader distribution in Atheliales, but a psilocybin cluster was not found.

**TABLE 1:** Summary statistics and psilocybin homolog counts for sequenced Atheliales species

Parameter	Data for:		
	*Athelia* sp. TMB TB5	*Fibularhizoctonia psychrophila* CBS 109695 (4)	*Piloderma croceum* F 1598 (5)
Genome size (bp)	79,838,449	95,125,689	59,326,866
No. of contigs	1,682	1,918	715
*N*_50_ (bp)	133,022	289,599	529,349
Contig *L*_50_	165	98	33
GC content (%)	51.76	51.67	46.28
Complete BUSCOs (%)	97.60	98.00	97.30
No. of predicted genes	22,782	32,946	21,576
Mean gene length (bp)	1,834.04	1,467.99	1,451.82
Proportion of assembly covered by annotation (%)	52.79	50.84	52.82
No. of genes in orthogroups	20,595	29,040	16,266
No. of psilocybin decarboxylase (PsiD) close homologs	2	2	0
No. of psilocybin hydroxylase (PsiH) close homologs	15	28	0
No. of psilocybin kinase (PsiK) close homologs	8	6	0
No. of psilocybin methyltransferase (PsiM) close homologs	0	1	1

### Data availability.

The assembly and annotation of *Athelia* sp. TMB strain TB5 were deposited in GenBank under accession number JACXVL000000000, with SRA accession numbers PRJNA641386, SRR12880626 (MinION read library), and SRR12880627 (Illumina read library).
